# Leg compression for preventing hypotension after spinal anesthesia in elderly hip fracture patients

**DOI:** 10.1007/s00590-026-04667-4

**Published:** 2026-02-02

**Authors:** Noratep Kulachote, Panupong Chatareeyagul, Norachart Sirisreetreerux, Nachapan Pengrung, Theerawat Chalacheewa, Amorn Vijitpavan, Paphon Sa-ngasoongsong

**Affiliations:** 1 Department of Orthopedics, Mahidol University, Bangkok, Thailand; 2 Department of Anesthesiology, Mahidol University, Bangkok, Thailand

**Keywords:** Perioperative hypotension, Intraoperative hypotension, Postoperative hypotension, Compression stocking, Postoperative complication

## Abstract

**Purpose:**

Hip fracture (HF) is a common geriatric trauma resulting in a substantial rate of mortality and morbidity. Previous studies have shown that the application of leg compression significantly reduced the incidence of perioperative hypotension (PeH) and hypotension-related complication in obstetric surgery. The objective of this study was to evaluate the effect of medical compression stocking for prevention of PeH in elderly patients undergoing HF surgery.

**Methods:**

Sixty patients were randomized in 2 groups: compression stocking (CS) group and no compression stocking (NCS) group (*n* = 30 each). Compression stockings were worn on the uninjured leg after spinal anesthesia and then removed after 24 h postoperatively. Demographic and perioperative data were collected. Primary outcome was the incidence of PeH measuring as 3 methods: intraoperative hypotension (IoH), postoperative hypotension (PoH), and concomitant intraoperative and postoperative hypotension (CoIPH). The secondary outcomes were the incidence of using intraoperative vasopressor and in-hospital hypotension-related complications related to hip fracture.

**Results:**

Baseline characteristics such as age, gender, diagnosis and operation were not significant difference between groups (*p* > 0.05 all). Regards the PeH, CS group demonstrated a significantly lower incidence in CoIPH than NCS group (0 patients vs. 6 patients, *p* = 0.02), and also showed a non-significantly lower incidence of the PoH and hypotension-related complication (*p* = 0.13 and 0.10, respectively). However, the IoH, and the need of intraoperative vasopressor did not significantly differ between both groups (*p* > 0.05 all).

**Conclusion:**

The application of medical compression stocking in elderly patients undergoing HF surgery demonstrated an ability to reduce the incidence of hypotension perioperatively and might prevent in-hospital hypotension-related complication.

## Background

 Perioperative hypotension (PeH) during hip fracture (HF) surgery is a common problem as the incidence being high as 37.9–79.8% [1–3]. Previous studies demonstrated that PeH significantly associated with postoperative morbidity and mortality, especially in the elderly patients [4–6], such as postoperative delirium, stress ulcer or gastrointestinal bleeding, acute kidney injuries, myocardial ischemia and stroke [7]. The causes of PeH could be related to the patients, anesthesia, and surgery. The patient-related factors included advanced age, anemia, low blood pressure before anesthesia induction, hypovolemia, high grade American Society of Anesthesiologists (ASA) status, previous stroke, and chronic use of antihypertensive drugs [3, 6, 8–10]. PeH usually occurred after anesthesia induction, especially for general and spinal anesthesia with an incidence varied from 8% to 90% [5, 11, 12].

In HF surgery, intraoperative hypotension (IoH) usually occurred after spinal anesthesia, as same as in caesarean section, due to the arterial and venous vasodilatation resulting from the sympathetic block along with a paradoxical activation of cardioinhibitory receptors [13]. Previous studies in caesarean section revealed that leg compression with elastic bandage was as an effective method for reducing the incidence of hypotension after spinal anesthesia [14–17], with a significant reduction in arterial blood pressure on during the first 15-minute period after spinal anesthesia [18]. Leg compression could reduce IoH by improving venous return and blood flow to the heart, particularly after anesthesia induction and often used with other measures such as vasopressors [19]. However, to the best of our knowledge, there is no evidence of leg compression in HF surgery would be useful for prevention of PeH. Our hypothesis is leg compression during perioperative period of HF surgery with a medical compression stocking would decrease the incidence of PeH and postoperative complications related to hypotension.

The aim of this study is to (1) evaluate the effect of compression stockings on the uninjured leg during the HF surgery on the PeH, IoH, postoperative hypotension (PoH) and its related complications, and (2) correlate the type of hypotension with postoperative complications and identify the risk factors of hypotension.

## Materials and methods

### Study design and participants allocation

This study was a prospective, non-blinded, randomized control trial with 1:1 allocation ratio in a medical university hospital in (Ramathibodi Hospital, Mahidol University). Prior approval was obtained from the institutional review board (Protocol number ID xx-xx-xx). Informed consent was obtained from all patients who participated in this study, after the surgery was scheduled, in accordance with the Declaration of Helsinki. The manuscript was prepared according to the Consolidated Standards of Reporting Trials (CONSORT) guideline.

Eligible patients were those who were diagnosed as HF and scheduled for surgery within 48 h after admission between March 2017 and September 2018. Inclusion criteria were the patients that (1) aged over 60 years, (2) had normal preoperative coagulogram, (3) were planned for standard spinal anesthesia, and (4) had willing to participate and gave their informed consent. The exclusion criteria were those who had (1) severe peripheral vascular disease, (2) peripheral neuropathy, (3) cellulitis at the leg, (4) uncontrolled congestive heart failure, (5) deep vein thrombosis, and (6) allergy to compression stocking.

### Sample size calculation

Sample size was calculated using Power & Sample size (PS) calculation software and data review from 50 patients underwent HF surgery (prevalence PeH as 64%). We determined that a total of 50 patients would be required to provide an 80% power and type-1 error as 0.05, with the effect of 60% difference in PeH. To prevent the lost to follow-up during the study, a 20% dropout rate was added, resulting in the total amount of 60 patients.

### Randomization

**Fig. 1:**
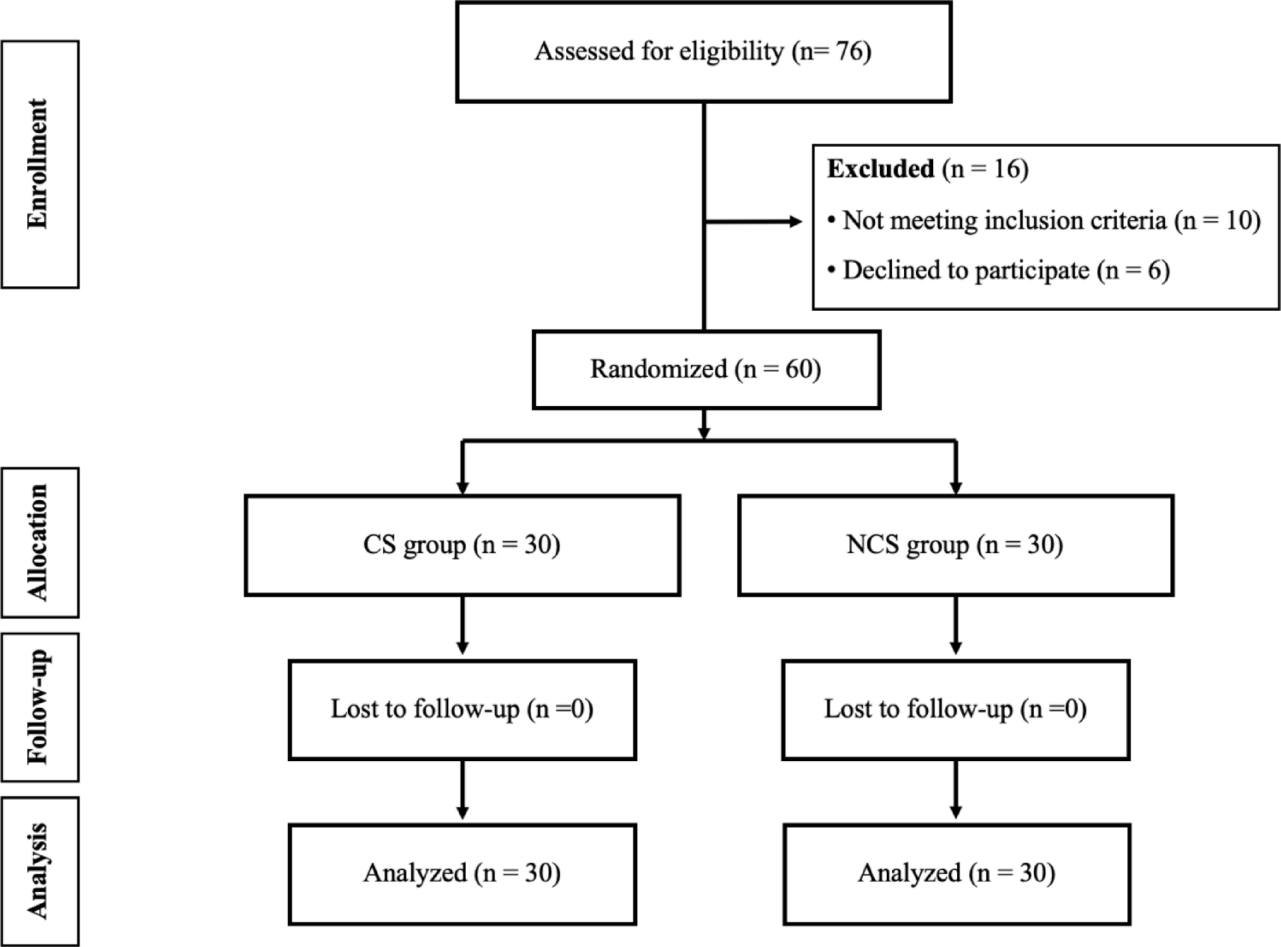
The flow diagram of this study

A randomization sequence was generated by STATA software (Stata Corp, College Station, Texas, USA), with block size of 6, and further concealed with sealed envelopes in the sequentially numbered container. The envelopes were sequentially opened intraoperatively, after successful spinal anesthesia, by research nurse who did not involve in the outcome assessment. Then all patients were randomized into 2 groups: compression stockings (CS) group and no compression stockings (NCS) group (30 patients each group) (Fig. 1).

### Study protocol

**Fig. 2:**
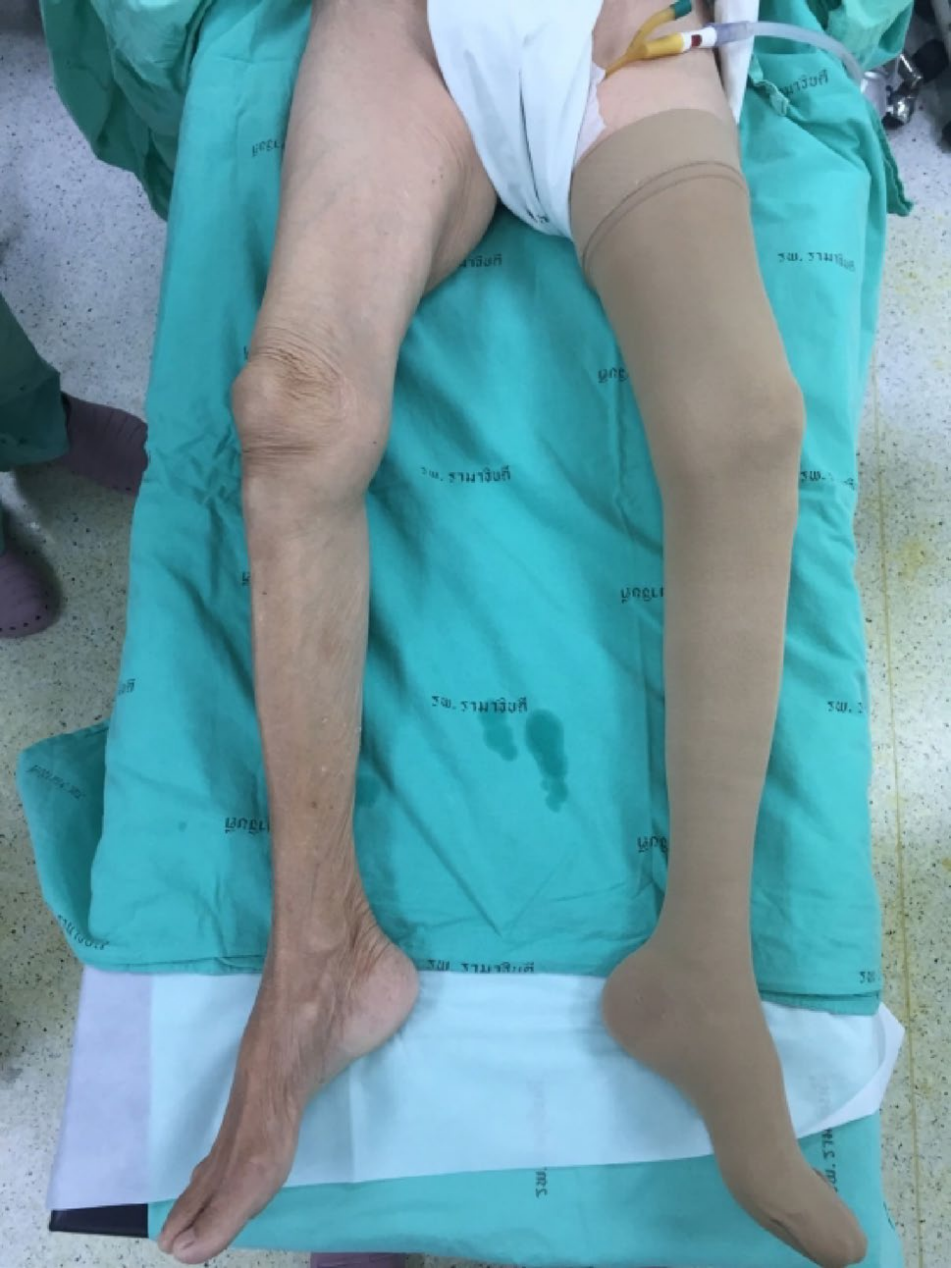
Demonstration of the medical compression stockings application after spinal anesthesia

After admitting into inpatient ward and giving their informed consent, all patients were treated with a standard perioperative pain management and hemodynamic optimization protocol. Oral regular acetaminophen 500 mg every 6 h were given until they were discharged. In-hospital rescue medication was intravenous morphine 3 mg every 4 h if 10-point visual analog scale ≥ 4. Preoperative hemodynamic optimization and monitoring included fluid resuscitation with crystalloid fluid to compensate for blood loss and dehydration, correction of electrolyte imbalance, and oxygen supplementation if needed. Vital signs, oxygen saturation, and urine output were assessed every 4 h. Postoperative daily hemoglobin level was routinely measured in the morning. Packed red cell transfusion was considered in those with hemoglobin level < 8.0 g/dL and those with signs of ischemia (dyspnea, hemodynamic instability) [20]. The primary goals were stable vital signs with a target of urine output > 0.5 mL/kg/hour. All patients underwent early HF surgery (within 48 h) with the standard spinal anesthesia by one of two authors (TC, AV). Afterward, the sealed opaque envelopes were opened and then the patients were randomized into 2 groups: CS and NCS group. Regards CS group, a medical compression stocking (JOBST^®^ UltraSheer, BSN medical GmbH, Hamburg, Germany) corresponding to the leg size with 30–40 mmHg pressure was applied on an uninjured leg (Fig. 2), while none of device was applied in the NCS group. All operations were performed by one of authors (PS, NK, NS, and NP) who had experience in hip fracture surgery more than 10 years. Non-displaced femoral neck fractures were treated by multiple screw fixation, and displaced femoral neck fractures were treated by total hip replacement in physically active and medically fit patients or bipolar hip replacement in senile inactive patients with low functional demand. Intertrochanteric fractures were treated by dynamic hip screw in stable and noncomminuted fracture pattern or proximal femoral nail anti-rotation in unstable fracture pattern. Vital signs were recorded sequentially at 1, 5 and 10 min before operation. During the perioperative period, vital signs were recorded every 5 min until the operation was completed. During the postoperative period, vital signs were recorded every 5 min, 30 min and 1 h on the first day and compression stocking is removed after 24 h. Vital signs were then recorded every 4 h after the first day until the 4th day or until the patient was discharged.

### Outcome assessment

Baseline characteristics were collected. Primary outcome was the incidence of perioperative hypotension measuring with 3 methods: intraoperative hypotension (IoH), postoperative hypotension (PoH), and concomitant intraoperative and postoperative hypotension (CoIPH). Hypotension was defined as mean arterial blood pressure < 60 mmHg and/or systolic blood pressure < 90 mmHg or decrease more than 80% of baseline within 24 h after surgery, and lasting more than 10 min, or vasopressor was administered [4, 9]. IoH was defined as hypotension during operation, PoH was defined as hypotension after operation with 24 h postoperatively. CoIPH was defined as hypotension that occurred in both intraoperatively and postoperatively.The secondary outcomes were the incidence of using intraoperative vasopressor, blood transfusion, and hypotension-related complications during hospital admission. Hypotension-related complication was defined as end organ injury associated with hypotension including mortality, stroke, myocardial infarction, cardiac arrythmia or arrest, and acute kidney injury [21].

### Statistical analysis

Continuous data with normal distribution were presented as means and standard deviations and compared using t-tests. Categorical data were presented as proportions and compared using Fisher’s exact test. Relative risk (RR) and 95% confidence interval (CI) were provided as a measure of effect. Correlation between each type of hypotension and hypotension-related complication was calculated with regression analysis and presented with RR and 95% CI. To assess risk factors for hypotension, univariate and multivariable logistic regression analyses were performed to determine associations between risk factors and outcomes. A p-value < 0.1 was used to identify potential predictive factors, with statistical significance defined as *p* < 0.05. Data analysis was performed by using MedCalc software.

## Results

During the study period, 76 patients with elderly hip fracture were recruited. Sixteen of them were excluded due to severe peripheral vascular disease (*n* = 1), peripheral neuropathy (*n* = 8), deep vein thrombosis (*n* = 1), and unwilling to participate (*n* = 6). Therefore, a total of 60 patients were randomized in 2 groups (30 each group): CS and NCS group. Regards the demographic data of all patients, the average age was 76.3 year (range 60–92 year). Forty-eight of them (80.0%) were female. The most common comorbid disease was hypertension (68.3%). There were 40 patients (66.7%) sustained femoral neck fracture, while 20 patients (33.3%) had intertrochanteric fracture. The average preoperative hemoglobin was 11.3 g/dL (range 8.0–14.9 g/dL). Those with femoral neck fracture were treated with either multiple screw fixation (*n* = 2, one each group), total hip replacement (*n* = 2, one each group) or bipolar hip replacement (*n* = 36, 18 in CS group and 20 in NCS group). Those with intertrochanteric fracture were treated with proximal femoral nail anti-rotation (*n* = 20, 11 in CS group and 9 in NCS group). Table 1 showed baseline characteristics data between both groups. No significant difference on their demographic data was found between both groups. However, although there was non-significant in type of operation between both groups, the patients underwent cemented arthroplasty in CS group (*n* = 8) were lower than NCS group (*n* = 16).

**Table 1:** Baseline characteristics

	CS group (*n* = 30)	NCS group (*n* = 30)	*p*-value
Age (year)^a^	76.7 ± 9.1	76.0 ± 9.0	0.75
Gender (female: male) ^b^	23:7	25:5	0.32
BMI (kg/m^2^)^a^	22.5 ± 4.9	23.1 ± 6.4	0.41
Frature side (right: left)^b^	12:18	15:15	0.44
Underlying disease^c^			
Hypertension	21	20	1.00
Diabetes	11	7	0.40
Ishemic heart disease	4	2	0.67
Cerebrovascular disease	3	2	1.00
Chronic kidney disease	4	3	1.00
Antiplatelet use^c^			
Aspirin	5	2	0.42
Clopidogrel	2	2	1.00
Fracture location (FN/IT) ^b^	19:11	21:9	0.58
Operation^b^			
Multiple screw fixaiton	1	1	0.14
Proximal femoral nail anti-rotation	11	9	
Cemented arthroplasty	8	16	
Cementless arthroplasty	10	4	
Operative time (minute)^a^	124.0 ± 22.2	125.8 ± 2.5	0.81
Preoperative Hb (g/dL)^a^	11.3 ± 1.9	11.4 ± 1.5	0.86
EBL (ml) d	200 (50–250)	175 (100–400)	0.69

^a^value presented as mean ± standard deviation, ^b^value presented as ratio of number of cases, ^c^value presetned as number of cases, ^d^value presented as medain (range)

BMI, body mass index; FN, femoral neck; IT, intertrochanter; Hb, hemoglobin; EBL, estimated blood loss

Table 2 demonstrated the change in mean arterial blood pressure throughout intraoperative period and postoperative period for 3 days, and the use of vasopressor in both groups. During the intraoperative and postoperative period, there was no significant difference in mean arterial blood pressure and use of vasopressor between groups (*p* > 0.05 all).

**Table 2:** Change in mean arterial blood pressure during intraoperative and postoperative period

	CS group (*n* = 30)	NCS group (*n* = 30)	*p*-value
Intraoperative period			
Before induction	109.4 ± 17.3	114.6 ± 18.9	0.28
After induction			
15 min	79.7 ± 14.3	85.7 ± 14.9	0.12
30 min	78.8 ± 12.7	83.9 ± 17.8	0.20
45 min	79.3 ± 12.5	81.1 ± 15.3	0.64
60 min	84.3 ± 13.4	87.8 ± 15.3	0.39
75 min	82.4 ± 16.6	84.7 ± 13.5	0.60
90 min	83.4 ± 13.3	84.2 ± 14.1	0.86
105 min	85.8 ± 14.2	91.6 ± 18.3	0.31
120 min	83.2 ± 14.9	87.6 ± 13.7	0.41
Vasopressor use	15 (50%)	15 (50%)	1.00
Postoperative period			
POD0			
At ward	86.3 ± 14.6	87.2 ± 12.9	0.80
6 h	84.1 ± 11.6	88.1 ± 12.8	0.36
POD1	81.4 ± 8.3	79.9 ± 10.8	0.58
POD2	80.6 ± 10.8	80.1 ± 9.6	0.85
POD3	82.4 ± 10.0	82.2 ± 13.7	0.96

min, minute, POD, postoperative day

Incidence of hypotension, blood transfusion and postoperative complications were shown in Table 3. In terms of hypotensive events, CS group show a non-significant lower incidence of IoH and PoH compared to NCS group (*p* = 0.60 and 0.13, respectively). However, the incidence of CoIPH in CS group was significantly lower than those in NCS group (0% vs. 20%, *p* = 0.02). Regards the postoperative complication, one patient in CS group developed complication as congestive heart failure with acute kidney injury while 6 patients in NCS group experienced postoperative complications (1 stroke, 1 acute kidney injury, 1 myocardial infarction with acute kidney injury, 1 myocardial infarction, 1 cardiac arrest, and 1 atrial fibrillation (*p* = 0.10).

**Table 3:** Outcomes related to hypotension

	CS group (*n* = 30)	NCS group (*n* = 30)	*p*-value ^a^	RR (95%CI)
Incidence of hypotension				
IoH	8 (30%)	11 (36.7%)	0.60	0.73 (0.34 to 1.55)
PoH	4 (13.3%)	10 (33.3%)	0.13	0.40 (0.14 to 1.14)
CoIPH	0 (0.0%)	6 (20.0%)	0.02*	0.08 (0.00 to 1.31)
Blood transfusion	5 (16.7%)	7 (23.3%)	0.75	1.09 (0.84 to 1.40)
Complications	1 (3.3%)	6 (20.0%)	0.10	0.17 (0.02 to 1.30)

IoH, intraoperative hypotension, PoH; postoperative hypotension, CoIPH, concomitant intraoperative and postoperative hypotension

^a^*p*-value calculated from Fisher Exact test; RR, relative risk; CI, confidence interval

*Significant difference with *p*-value < 0.05

Table 4 demonstrated the correlation between hypotension-related postoperative complication and type of hypotension. Although PeH, IoH, and CoIPH did not significantly associated with hypotension-related postoperative complication, there was a significant correlation between PoH and hypotension-related postoperative complication (odds ratio [OR] 12.22 (95% confidence interval [CI] 2.04 to 73.19), *p* = 0.01).

**Table 4:** Correlation between hypotension-related postoperative complication and type of hypotension

	Odds ratio (95% confidence interval)	*p*-value
PeH	3.52 (0.63 to 19.84)	0.15
IoH	0.85 (0.15 to 4.82)	0.85
PoH	12.22 (2.04 to 73.19)	0.01*
CoIPH	4.90 (0.71 to 33.79)	0.11

PeH; perioperative hypotension, IoH; intraoperative hypotension, PoH; postoperative hypotension, CoIPH; concomitant intraoperative and postoperative hypotension

*Significant differnce with *p* < 0.05

Table 5 showed the logistic regression analysis of perioperative risk factors associated with IoH and PoH. From univariate analysis for IoH, the vasopressor administration was the only one significant predictor. Therefore, multivariate analysis did not perform. Regarding to PoH, univariate analysis showed that four risk factors—including intertrochanteric fracture, use of intramedullary nail, cemented arthroplasty, and use of leg compression—were predictors for PoH with *p* < 0.1. However, multivariate analysis revealed that cemented arthroplasty was the only one independent factor for PoH (OR 6.35, 95%CI 1.69 to 23.88, *p* = 0.01).

**Table 5:** Logistic regression analysis for intraoperative hypotension (IoH) and postoperative hypotension (PoH)

	IoH				PoH			
	Univariate analysis		Multivariate analysis		Univariate analysis		Multivariate analysis	
	OR (95% CI)	*p*-value	OR (95% CI)	*p*-value	OR (95% CI)	*p*-value	OR (95% CI)	*p*-value
Age	0.99 (0.93 to 1.05)	0.68			1.01 (0.94 to 1.08)	0.8		
Female gender	1.29 (0.30 to 5.54)	0.73			0.77 (0.17 to 3.41)	0.73		
Intertrochanteric fracutre	1.26 (0.40 to 3.93)	0.7			0.26 (0.05 to 1.30)	0.1		
Hypertension	1.98 (0.63 to 6.22)	0.24			1.90 (0.55 to 6.57)	0.31		
Chronic kidney disease	3.09 (0.34 to 27.63)	0.31			1.95 (0.21 to 17.73)	0.55		
Preoperative hemoglobin	0.96 (0.66 to 1.39)	0.82			1.14 (0.74 to 1.75)	0.55		
mABP before induction	0.99 (0.96 to 1.02)	0.55			1.02 (0.99 to 1.05)	0.22		
Use of vasopressor	10.29 (2.56 to 41.37)	0.001*			1.45 (0.44 to 4.86)	0.54		
Intramedullary nail	1.26 (0.40 to 3.93)	0.7			0.26 (0.05 to 1.30)	0.1		
Cemented arthroplasty	0.91 (0.30 to 2.80)	0.87			6.35 (1.69 to 23.88)	0.01*	6.35(1.69 to 23.88)	0.01*
Leg compression	0.63 (0.21 to 1.88)	0.41			0.31 (0.08 to 1.13)	0.08		
Operative time	1.00 (0.98 to 1.03)	0.81			1.02 (0.99 to 1.05)	0.34		
Estimated blood loss	1.00 (1.00 to 1.00)	0.18			1.00 (1.00 to 1.00)	0.7		
PRC transfusion	0.87 (0.28 to 2.69)	0.81			0.26 (0.03 to 2.01)	0.2		

* indicates significant difference with p<0.05

## Discussion

In the present study, the incidences of perioperative hypotension as intraoperative hypotension (IoH), postoperative hypotension (PoH), and concomitant intraoperative and postoperative hypotension (CoIPH) were 32%, 23%, and 10% respectively. Our findings are compatible with previous studies [1–4]. Jitsinthunun et al. showed the incidence of perioperative hypotension in elderly hip fracture Thai patients underwent spinal anesthesia was 38% [10]. Regarding to the incidence of PeH in other anesthesia techniques such as general anesthesia and peripheral anesthesia, previous studies showed that the prevalence of PeH in general anesthesia was also high as ranging from 8% to 68% [5, 11]. Messina et al. conducted a prospective randomized controlled trial in 20 hip fracture patients and revealed that the spinal anesthesia provided a more stable hemodynamic profile than general anesthesia. However, despite both groups were experienced hypotension after induction, the etiology of hypotension was different. While hypotension from spinal anesthesia was mainly from the reduction in systemic vascular resistance index, those from general anesthesia was primarily to the reduction in stroke volume index and cardiac index [1]. Additionally, Mounet et al. also demonstrated the incidence of hypotension in the hip fracture patients with intermediate risk for surgery was significantly higher in general anesthesia (90%) than continuous spinal anesthesia (54%) and multiple nerve blocks (51%) [12].

Our results demonstrated the effectiveness of leg compression with medical compression stockings on the uninjured leg in hip fracture surgery under spinal anesthesia by reducing the IoH and PoH as 6.7% and 20.0%, respectively. Although the difference in IoH and PoH were not significantly differ, the incidence of CoIPH in the patients with leg compression (CS group) was significantly lower compared control group (NCS group) (0% vs. 20%, *p* = 0.02, relative risk 0.08 [95%CI 0.00 to 1.31]) (Table 3). This could be explained by the prolonged effect of leg compression from intraoperative period to 24 h postoperatively, by medical compression stocking, for preventing hypotension after spinal anesthesia in elderly patients who underwent HF surgery. Therefore, those who have risk of PeH might be beneficial for this method as same as those in previous studies with caesarean sections [14, 15]. These benefits could be seen in the reduction of postoperative complication by 17% as shown in Table 3. However, our positive findings might be different from those in previous studies with caesarean sections [14, 15]. due to the difference in age and comorbid diseases, type of operation, and applicability of one-leg compression technique. Although, the results of this study were implied to the hip fracture surgery under spinal anesthesia, those under other anesthesia techniques such as general anesthesia and peripheral anesthesia would also have benefits from leg compression due to the ability of increasing blood volume into the central circulation. Moreover, previous study by Park et al. had shown a significant reduction in the incidence of post-induction hypotension after general anesthesia in the elderly patients underwent robotic-assisted laparoscopic prostatectomy by using pneumatic leg compression before induction of general anesthesia compared to the control group (10% vs. 58%, respectively) [22].

In addition, the results of this study also revealed the significant correlation between PoH and postoperative complications related to hypotension in elderly undergoing HF surgery (relative risk 12.2, 95% confidence interval 2.0 to 73.2, *p* = 0.006), as same as in previous studies [21, 23]. These findings signify the importance of perioperative management during HF surgery for prevention of PeH [2, 3, 23–25].

The present study also had some limitations. Firstly, although this study was designed as a prospective randomized controlled trial, the sample size was relatively small for identify the effect of leg compression on elderly HF patients, especially in those who had multiple comorbid disease or had HF surgery with other anesthetic techniques such as general anesthesia or peripheral anesthesia. Secondly, some important outcomes, such as cardiac output, was not collected due to the invasiveness and risk of complication from the measurement method as using pulmonary artery catheter. Lastly, our study did not explore the effect of the leg compression with other type of medical compression stockings with different pressure or using elastic bandage as same as the previous studies in caesarean section. Therefore, the future studies with large sample size and different protocol are needed.

## Conclusion

The application of leg compression by using medical compression stocking on the uninjured leg in hip fracture surgery with spinal anesthesia showed an ability to reduce the incidence of perioperative hypotension and might prevent in-hospital hypotension-related complication. We recommend using the leg compression in the elderly patients undergoing HF surgery, especially those who had high risk for postoperative complication.

## Data Availability

No datasets were generated or analysed during the current study.
